# Illicit substances in school-confiscated vapes: Lessons from a laboratory-driven public health pilot

**DOI:** 10.1016/j.puhip.2026.100770

**Published:** 2026-03-15

**Authors:** H. Sharrod-Cole, J. Danks, C. Holdcroft, R. Patel, L. Starbrook

**Affiliations:** Black Country Pathology Services, Royal Wolverhampton NHS Trust, Wolverhampton, UK

**Keywords:** Vaping, Synthetic cannabinoids, School confiscations, Illicit substances, Public health surveillance, Spice

## Abstract

**Objectives:**

Youth vaping in UK schools has escalated, with reports of vapes sold as THC products containing synthetic cannabinoid receptor agonists (SCRAs) and other illicit substances, prompting health emergencies. This pilot study aimed to determine the prevalence and types of illicit substances present in vapes confiscated from secondary schools to inform future public health surveillance and interventions.

**Study design:**

Cross-sectional pilot study of confiscated vaping products collected from secondary schools in a defined UK region.

**Methods:**

In May 2025, 80 secondary schools in the Black Country and West Birmingham were invited to donate confiscated vapes. Seven schools participated, and 212 vape products were collected in July 2025. LC-QTOF-MS screened for illicit substances, with confirmatory LC-MS/MS for SCRAs.

**Results:**

Confiscated devices comprised 161 disposable vapes (76%), 32 refillable devices, and 19 vape liquids (mean 30.1 per school; 95% CI 17.9–42). Of these, 204 samples were analysed, 14 samples from six schools contained illicit substances (6.9%): SCRAs were dominant (MDMB-4en-PINACA n = 13), plus 4F-MDMB-BINACA (n = 4), 5F-ADB (n = 2), MDMB-PINACA (n = 1); one liquid had all four SCRAs plus ecstasy, ketamine, mephedrone and ethcathinone. Half of positive vapes were disposable; the remaining seven included six were refillable and one liquid.

**Conclusions:**

Illicit substances, particularly SCRAs also known as Spice, were detected in vapes confiscated from schools. SCRAs are frequently confused with natural cannabis. SCRAs are not natural cannabinoids but more potent and dangerous synthetic compounds. Improved awareness and understanding of their potential harms are critical for informing public health messaging and safeguarding young people. Multi-agency collaboration and laboratory surveillance of confiscated items are essential to enable targeted education.

## 1 What this study adds

• First pilot in the Black Country and West Birmingham identifying presence of illicit drugs in school-confiscated vapes.
• Highlights dominance of synthetic cannabinoids with low prevalence of natural cannabinoids supporting possible mis-selling as cannabis.
• Demonstrates feasibility of laboratory driven surveillance for timely public health insights from opportunistic school collections.

## 2 Implications for policy and practice

• Urges multi-agency protocols for collating and testing seized/confiscated vapes to map local trends and inform rapid response.
• Supports targeted education, specifically around synthetic cannabinoids, and further research addressing purchase channels and mis-selling risks.
• Informs healthcare preparedness for synthetic cannabinoid toxicity, emphasising refillable device vigilance beyond disposable vape bans.

## 3 Introduction

The growing use of vaping products has become a major public health concern, not only due to their uncertain long-term health effects but also because of mounting evidence that some contain illicit or undeclared substances [1]. While electronic cigarettes are widely promoted as harm-reduction tools and show effectiveness in supporting smoking cessation [2], recent UK data suggest that a subset of these products deviate from legitimate nicotine replacement use [1]. Reports from Greater Manchester highlight the increasing detection of illegal drugs and novel psychoactive substances in unregulated vape liquids, particularly among young people [1]. Correspondingly, NHS Digital (2024) has reported rising hospital admissions linked to vaping-related disorders, underscoring the potential harms associated with adulterated or illicit vape products [3]. This is exemplified by reports of multiple cases of school-aged children requiring emergency medical support following the use of products sold as tetrahydrocannabinol (THC) vapes-the psychoactive component of cannabis [1,3]. During 2023/24, the Manchester drug analysis and knowledge exchange tested nine e-liquids/vapes that were purchased as THC vapes. All samples were linked to incidents in Greater Manchester high schools where one or more pupils had become unwell, resulting in ambulance callouts and in most cases, hospitalisation [3]. Instead of THC, these vapes were found to contain other harmful substances most notably synthetic cannabinoid receptor agonists (SCRAs). The Welsh Emerging Drugs and Identification of Novel Substances (WEDINOS) project has also found similar patterns, detecting SCRAs in 46% of e-liquid samples submitted between 2020 and 2021-most sold as cannabis-based products [4]

**Fig. 1:**
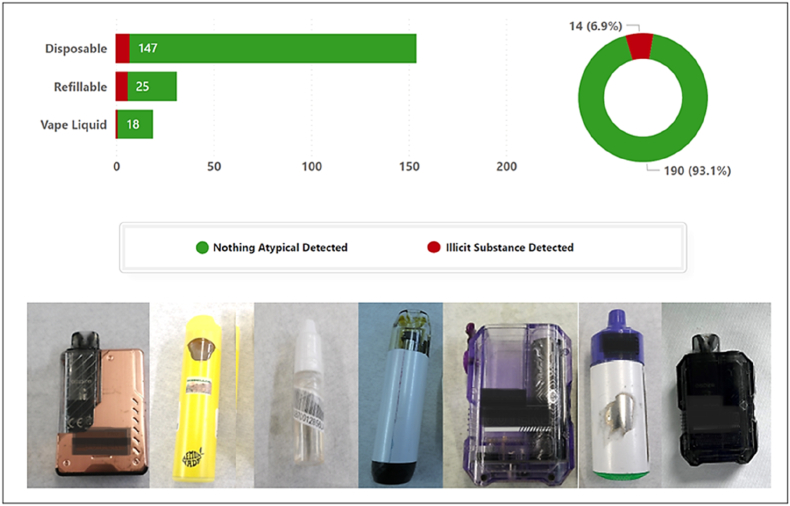
Analysis of vape samples for illicit substances The bar chart on the left shows the number of vape samples analysed, categorised by vape type. The pie chart on the right illustrates the percentage of analysed samples testing positive for illicit substances. The photographs underneath depict a selection of vapes confirmed to contain illicit substances.

SCRAs, sometimes called “Spice” or “K2” are psychoactive compounds designed to mimic natural cannabis but are often significantly more potent and unpredictable. They have been associated with drowsiness, coma, agitation, seizures and psychosis. [5,6]. A recent study highlighted the growing prevalence of “Spice” in vapes used by UK schoolchildren, raising alarms about the covert nature of these substances and the lack of awareness among users [7].

In our hospital Trust in Sandwell and West Birmingham NHS Trust, toxicological testing has identified MDMB-4en-PINACA, a potent SCRA, in the urine of several patients, including adolescents and young adults, who presented to the ED with symptoms following exposure to the SCRAs through vaping [6].

University of Bath research demonstrates widespread access to illicit vapes containing synthetic cannabinoids like Spice via social media platforms, where they can be purchased for a few pounds [7]. Due to this universal access via social media platforms, we hypothesise that the widespread contamination of vapes with illicit substances like Spice-previously documented in Greater Manchester, Wales, and other UK regions-is similarly prevalent in the Black Country. The aim of this pilot was therefore to test confiscated vapes from Black Country schools for the prevalence of such illicit substances in vapes used by young people locally, adding to the growing body of evidence and enhancing national data to drive urgent public health responses.

## 4 Methods

In May 2025, safeguarding leads and headteachers of 80 secondary schools across the Black Country and West Birmingham-representing the local population-were contacted by email to invite voluntary participation in this pilot study. Schools were asked to anonymously donate vapes confiscated from students for laboratory testing (individual donor numbers are not known). Sample collection was opportunistic, so no further information on population size, demographics, or other factors was sought, as no reliable relationships could be established or conclusions drawn; sample numbers were capped due to funding limitations. Responses were received from 12 schools, with seven continuing with donation. Vapes were collected from these seven schools in July 2025- most had been confiscated in the previous school term.

Liquid chromatography quadrupole time-of-flight mass spectrometry (LC-QTOF-MS) was used to screen samples for illicit substances by separating and identifying them via exact mass and structure. Suspected SCRAs underwent confirmatory analysis by a second method-liquid chromatography tandem mass spectrometry (LC-MS/MS) for high-confidence identification.

## 5 Results

A total of 212 confiscated vaping products were collected from seven schools (mean 30.1 per school; 95% CI 17.9-42). These comprised 161 disposable devices, 32 refillable devices, and 19 vape liquids. Of these, 204 samples yielded sufficient liquid for analysis.

Illicit substances were detected in 14 vapes (6.9%) from six of the seven participating schools. Among these, seven (50%) were disposable devices, five of which were identifiable by brand. The remaining samples included six refillable vapes and one vape liquid, meaning 6 of 31 refillable devices (19.4%) contained illicit substances compared with 7 of 161 disposable devices (4.3%) (see Fig. 1).

Illicit substances including four different SCRAs were detected across 13 samples: MDMB-4en-PINACA (most frequent; n = 13; present alone in eight and co-detected in five), 4F-MDMB-BINACA (n = 4), 5F-ADB (n = 2), and MDMB-PINACA (n = 1). A single vape liquid contained four SCRAs, along with additional illicit substances including MDMA (ecstasy), ketamine, mephedrone, and ethcathinone. The natural cannabinoids-cannabidiol, cannabigerol, cannabinol, Δ^8^-THC and Δ^9^-THC- were detected in a single sample.

The presence of SCRAs was not identifiable by any labelling or packaging, while the vape containing natural cannabinoids was clearly labelled as such (see Fig. 1).

Results from this pilot were promptly shared with participating schools for educational purposes, alongside local public health and clinical teams. Two important anecdotal insights emerged: first, a clear lack of understanding among safeguarding leads and schools regarding the differences between natural and synthetic cannabinoids, including their distinct risks and health consequences; second, an apparent scarcity of targeted educational content addressing the presence and dangers of illicit substances in vapes.

## 6 Discussion

These findings demonstrate illicit substances in 6.9% of tested vaping products confiscated from secondary schools in the Black Country and West Birmingham, predominantly SCRAs alongside ketamine, MDMA/ecstasy, mephedrone, and ethcathinone. Illicit substances may stem from deliberate adulteration, counterfeits, or contamination-this pilot study cannot differentiate. These findings are supported by other small studies from multiple areas across the UK. Craft *et al.*(2025) identified 5F-MDMB-PICA (a potent SCRA) in seven disposable vapes illegally sold in the UK as cannabis products but designed to mimic regulated brands. [8] Comparable patterns of illicit substance detection have also been reported by Cozier *et al.*(2025) who identified SCRAs in e-cigarettes seized from English secondary schools. [7] Copeland (2025) highlighted multiple illicit substances (cocaine, ketamine, nitazenes and benzodiazepines) in illicit “THC vapes” *via* WEDINOS data. [[5], [10]] MDMB-4en-PINACA dominance in our pilot mirrors its prevalence the UK [9].

Despite comprising only 15% of the total sample, refillable devices contained a higher proportion of illicit substances (19.4%), compared to disposables (4.3%), underscoring persistent accessibility of vapes despite the introduction of new regulations.

This pilot study supports an alarming and growing body of evidence of SCRAs (Spice) and other illicit substance presence in vapes in schools. Significantly lower natural cannabinoid prevalence suggests pupils may be being mis-sold SCRAs as cannabis-a dangerous misconception. This suggestion is supported by survey data and investigations, where young people have reported unexpected adverse effects from supposed THC vapes, leading them to suspect that what they had consumed was not THC, and in some cases was confirmed to be Spice [1,3,10].

While UK legislation targets sale of single use vapes through regulated channels, it is unlikely to resolve refillable misuse and may exacerbate it. In addition, surveys and anecdotal reports confirm purchases often occur via Snapchat, TikTok, and other social media platforms, hindering traceability through transient listings and anonymity [1,10].

The limitations of this pilot, and necessary methodological workarounds are acknowledged. Firstly, funding restricted the work to a regional focus on secondary schools in the Black Country and West Birmingham only; although alignment with other data and purported purchasing routes indicate this pilot is likely to be generalisable nationally. Multi-site surveillance across England would be required and is called for, for confirmation of these findings. Secondly, sampling was opportunistic, based on schools' willingness to participate rather than random or proportional selection, potentially introducing selection bias. Thirdly, only confiscated vapes were analysed, which may not fully reflect overall prevalence among students, though findings align closely with other studies.

Multi-agency collaboration is essential for systematic collation of seized, donated, and confiscated vapes, coupled with laboratory-driven testing and surveillance to map illicit substance prevalence. This approach would generate critical data enabling health authorities to deliver targeted education and interventions, including direct messaging on the very social media channels where these adulterated products are likely being purchased. Although further work is required to ascertain purchasing intent and channels more precisely. Such integrated efforts will empower young people to make informed choices, mitigate risks from synthetic cannabinoids and other illicit exposures, and strengthen UK public health responses.

## 7 Plain English summary

Electronic cigarettes, or vapes, are often used to help people stop smoking, but their use among young people has become a growing concern. Many pupils are now using vapes not only for nicotine, but also for substances advertised as cannabis. Alarming reports from schools have described students becoming unwell after vaping, sometimes needing emergency medical care. Recent evidence suggests that some so-called “cannabis vapes” actually contain dangerous synthetic drugs that can cause serious harm.

This pilot project investigated what drugs were present in vapes confiscated from secondary school students in the Black Country and West Birmingham area. Seven schools took part, providing over 200 devices for testing using advanced laboratory methods. Around 7% of the vapes contained illegal substances, mainly synthetic cannabinoids-chemical compounds that mimic cannabis but are often far more powerful and unpredictable. Some devices also contained drugs such as ecstasy, ketamine and mephedrone. None of these dangerous ingredients were reflected on the packaging or product names, meaning users would have no idea what they were inhaling.

The findings confirm that some young people are unwittingly being exposed to potent, illegal substances through vapes. These results raise serious public health concerns and highlight the need for better education, surveillance, and testing of vapes found in schools. A national approach linking schools, laboratories, and health authorities will be vital to track emerging risks, identify harmful substances early, and help protect young people from unknown and potentially life-threatening exposures.

## Ethical statement

Formal ethical approval was not required for this study. Based on Health Research Authority guidance, this work is classified as health surveillance rather than research and therefore falls outside the scope of requiring ethics committee review.

## Funding

This pilot was funded by a charitable grant from the Robert Gaddie Memorial Fund, charity number 1016440.

## Declaration of competing interest

The authors declare that they have no known competing financial interests or personal relationships that could have appeared to influence the work reported in this paper.
